# Comparison of Recurrent Laryngeal Nerve Identification Time in the Lower Central Triangle during Thyroid Surgery Using Neurophysiological Mapping and Monitoring

**DOI:** 10.3390/medicina57080748

**Published:** 2021-07-24

**Authors:** Eunhye Lee, Keunchul Lee, Hyeong Won Yu, Su-jin Kim, Young Jun Chai, June Young Choi, Kyu Eun Lee

**Affiliations:** 1 Department of Surgery, Seoul National University Bundang Hospital, 82, Gumi-ro 173 Beon-gil, Bundang-gu, Seongnam-si 13620, Gyeonggi-do, Korea; eunhye.lee531@gmail.com (E.L.); curitty@gmail.com (K.L.); hyeongwonyu@gmail.com (H.W.Y.); 2 Department of Surgery, Seoul National University Hospital and College of Medicine, 101 Daehak-ro, Jongno-gu, Seoul 03080, Korea; su.jin.kim.md@gmail.com (S.-j.K.); kyueunlee@snu.ac.kr (K.E.L.); 3 Department of Surgery, Seoul National University Boramae Medical Center, 20 Boramae-ro 5-gil, Dongjak-gu, Seoul 07061, Korea; kevinjoon@naver.com

**Keywords:** thyroid, thyroidectomy, recurrent laryngeal nerve, intraoperative nerve monitoring, lower central triangle

## Abstract

*Background and Objectives:* Preserving the recurrent laryngeal nerve (RLN) is important in thyroid surgery. However, no standardized surgical method for locating the RLN has been established. We defined a new anatomical definition termed “lower central triangle” (LCT) for consistent identification of RLN and used intraoperative nerve monitoring (IONM) to aid in identification and dissection of RLN. *Materials and Methods:* Patients undergone thyroidectomy were reviewed retrospectively in Seoul National University Bundang Hospital from January to September 2017. Patients with papillary thyroid carcinoma, follicular neoplasm, and Graves’ Disease were included while right side non-RLN in arteriosus lusoria, cancer invasion, pre-existing vocal cord palsy, or under the age of 18 were excluded. RLNs were tested with IONM within LCT consisting of the lower pole as the apex and the common carotid artery as the opposite side. The samples were divided into two groups, IONM and non-IONM. *Results:* Forty lobes in total were included, 22 in IONM group and 18 in non-IONM group. Groups were not significantly different in age, cancer proportion, and accompanying thyroiditis while sex and nodule size differed. RLN detection time was 10.43 s shorter (*p* < 0.001), and confirmation time was 10.67 s shorter (*p* = 0.09) in IONM group than in non-IONM group. Both right and left RLNs were located predominately in the middle of LCT. No transient or permanent vocal cord palsy occurred. *Conclusions:* While IONM is an appropriate aid for thyroidectomy, our approach using LCT to locate the RLN is a novel definition of anatomy that provides prompt identification of the RLN in thyroid surgery.

## 1. Introduction

Preserving the recurrent laryngeal nerve (RLN) is a crucial aspect of thyroid surgery. Because the RLN is associated with vocal cord movement, intraoperative injury of the RLN can result in serious and debilitating complications. Unilateral injury of the RLN could compromise quality of life, manifesting as hoarseness, or potentially in incomplete closure of the glottis and subsequent dysphagia. On the other hand, bilateral RLN paralysis could be life-threatening, resulting in difficult extubation of the endotracheal tube or unavoidable tracheostomy in the worst case [1]. Most RLN injuries are transitory with or without intervention, but some remain permanent [2,3] and there are presently no available interventions to reverse the injury [4].

Preventative measures to preserve the RLN are therefore paramount to prevent injury. Precedent studies have suggested that routine identification of the RLN with fine dissection significantly reduces RLN palsy after thyroidectomy, and intraoperative nerve monitoring (IONM), a novel technology, further aids in identification and dissection of the RLN [5,6,7,8]. The use of IONM to identify the RLN during thyroid surgery significantly decreases transitory nerve palsy and tends to decrease permanent injury [9,10,11,12,13]. IONM is therefore becoming a gold standard for thyroid surgery and is now commonly used in high-volume centers [14,15,16].

Despite the pressing need to preserve the RLN in thyroid surgery, no specific surgical method has been established to isolate the RLN, as the path of the nerve varies between individuals [17]. Some representative methods previously suggested are a superior approach at the ligament of Berry or laryngeal entry, a lateral approach at the thyroid mid-pole within an RLN triangle consisting of the common carotid artery, trachea, and lower pole of the thyroid, and an inferior approach from the lowest point before RLN branching, the ima artery [18,19,20,21]. However, these approaches are not adjustable to every case and cannot be standardized, and a broadly standardized surgical protocol that can be applied in every case is urgently needed in the field.

Recurrent laryngeal nerve triangle, also known as Beahrs’ triangle, consists of recurrent laryngeal nerve, inferior thyroid artery, and common carotid artery. However, the RLN should be detected prior to the identification of the triangle and, thus, not practical in the operation.

**Figure 1:**
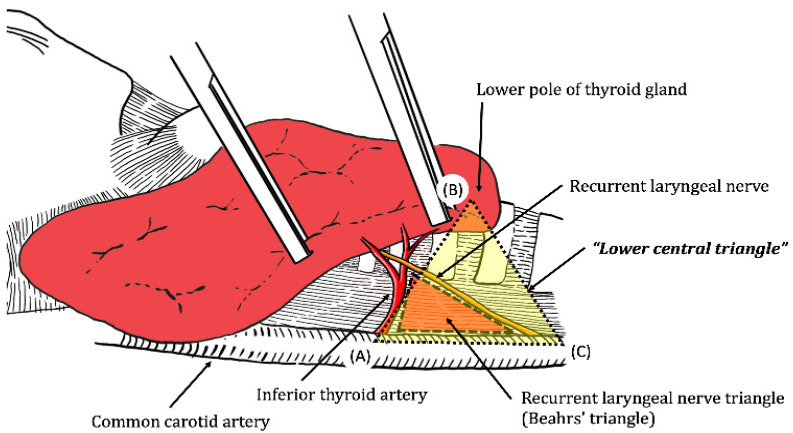
The constituents of lower central triangle (LCT). Viewed at the right lobe of thyroid with the lower pole of thyroid gland lifted.

In the present study, we defined a new anatomical definition, termed the “lower central triangle” (LCT) that allows identification of the RLN and enables effective use of the IONM. Lower central triangle consists of common carotid artery, inferior thyroid artery, and thyroid lower pole (Figure 1). The point (A) is where inferior thyroid artery crosses the common carotid artery, and the point (B) is the lower pole of thyroid when retracted medially while the point (C) is the most proximal point where the common carotid artery emerges underneath the clavicles. Lower central triangle is a surgical anatomy which is formed as the thyroid is retracted intraoperatively rather than a normal anatomy. RLN is placed at the median line of this triangle, a bisector of angle (C), providing a practical information to the operator.

The present study evaluates the efficacy of using IOMN to locate the RLN within the LCT during thyroid surgery. To do this, we measured the time-saving effect of IONM to localize and identify the RLN after exposing the LCT during surgery.

## 2. Materials and Methods

### 2.1. Patients

From January 2017 to September 2017, patients that underwent either total thyroidectomy or thyroid lobectomy in the Department of Surgery at Seoul National University Bundang Hospital were reviewed retrospectively. Patients that underwent thyroid lobectomy due to papillary thyroid carcinoma (PTC), follicular neoplasm (FN), or Graves’ disease were subject to review. Patients with right-sided non-RLN in arteriosus lusoria, vocal cord palsy in pre-operative laryngoscopy, cancerous invasion of the RLN, or under 18 years of age were excluded from analyses. Patient gender and age, malignant or benign nature of nodules, nodule sizes, and presence of thyroiditis were collected from the reviewed cases. The usage of IONM and surgical information such as the side of lobectomy and location of the RLN in the LCT were also collected. All surgeries were conducted by a single surgeon. Cases were divided into two groups, the IONM and non-IONM groups. The Institutional Review Board at the institution approved the study.

### 2.2. Procedure

A Nerve Integrity Monitor (NIM) was used for the IONM system. Electromyography (EMG) and an endotracheal tube size of 6.0 mm for women and 7.0 mm for men were used with cooperation of the Department of Anesthesiology [22].

The length of time (s) required for RLN identification after LCT dissection were recorded. In the IONM group, we defined “RLN mapping” as opening the space between the thyroid and carotid sheath and touching with a stimulating probe (current 2 mA) at the tracheoesophageal groove near the inferior thyroid artery and “RLN confirmation” as testing the localized RLN with a stimulation current of 1 mA to validate the nerve. The “mapping time” and “RLN confirmation time” defined as a time spent for dissection and re-check of RLN with IONM were measured.

As a comparable term for RLN mapping in the non-IONM group, “RLN detection time” was recorded as the gross time spent searching for the RLN. “RLN conformation time” was recorded as the amount of time required to check the course of the RLN toward the larynx.

### 2.3. Anatomical Definition of the Lower Central Triangle (LCT)

**Figure 2:**
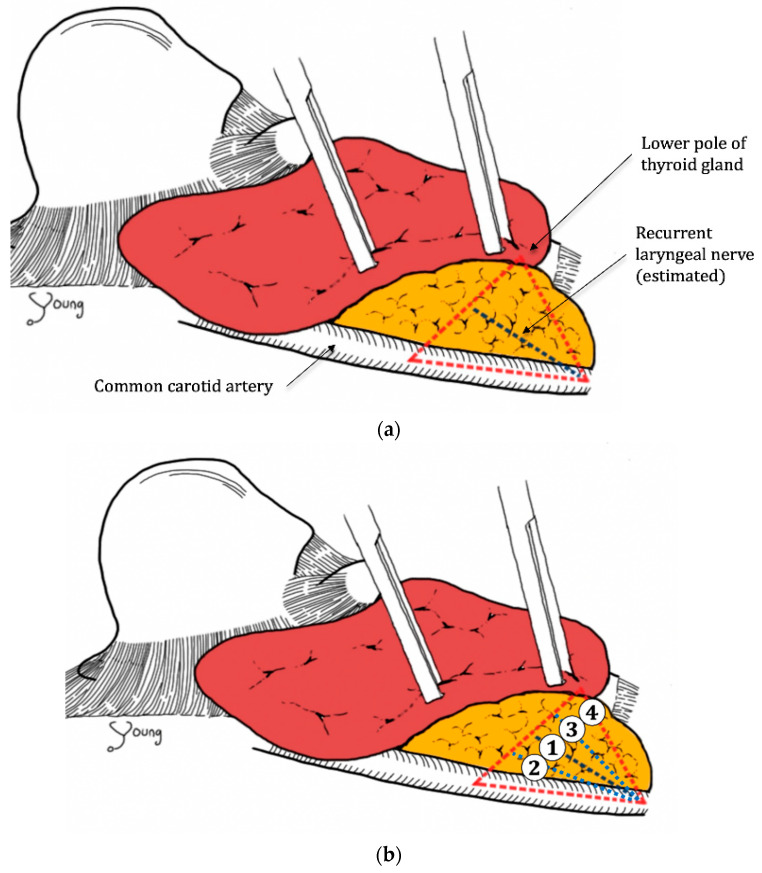
(**a**) The common carotid artery and lower pole of the thyroid gland form the “lower central triangle” (LCT), with the recurrent laryngeal nerve (RLN) lying in the middle of the triangle, viewed at the right lobe of thyroid with the lower pole of thyroid gland lifted. (**b**) Allocation of section numbers to characterize RLN location, viewed at the right lobe of thyroid with the lower pole of thyroid gland lifted.

The LCT, an imaginary anatomical definition for the modified inferior approach, consists of the common carotid artery, inferior thyroid artery, and the lower pole of the thyroid gland. The common carotid artery is the base side of the triangle, while the lower pole of the thyroid gland is a vertex opposite to the common carotid artery (Figure 2a). The RLN most commonly runs in the middle of this triangle (Figure 2b). The surgeon dissected into the carotid sheath, located the lower central triangle using the criteria above, and localized and identified the RLN with or without IONM.

To localize the RLN and analyze the frequency of its location, we divided the triangle into four sections and allocated a number (1–4) for each section. The numbers were assigned as shown in Figure 2b. Because we hypothesized that the nerve primarily ran through the middle of the triangle, we allocated number ‘1’ for the middle section. For the remaining sections, we allocated ‘2’ for the base of the triangle proximal to the common carotid artery, ‘4’ for the uppermost part of the triangle proximal to the lower pole of thyroid gland, and ‘3’ in between ‘2’ and ‘4’. We measured which section had the most cases for RLN localization. Both the right and left lobes were allocated with the numbers in the same order and included in analyses.

### 2.4. Statistics

Comparisons of characteristics between the non-IONM group and the IONM group were conducted with a Student’s *t* test for continuous variables and chi-square test for categorical variables. Mapping/detection time and identification time were analyzed with IONM with assessment by univariate analysis, and the comparison was performed with a Student’s *t* test. All statistical analyses were performed with IBM SPSS statistics 22 system (IBM, Armonk, NY, USA).

## 3. Results

Forty RLNs were examined, with 22 in the IONM group and 18 in the non-IONM group. Clinical characteristics are shown in Table 1. The mean age of the IONM group was 51.7 years, while the non-IOMN group was 51.8 years. The gender ratio of male to female was similar in both groups. The proportion of cancer cases in each group was similar, with 86.4% for the IONM group and 94.4% for the non-IONM group, and this difference was not statistically significant. Mean size of thyroid nodule was slightly bigger in the IONM group (2.22 cm) than in the non-IONM group (1.21 cm, *p* = 0.037). The final pathology review revealed that 45.5% of the IONM group was accompanied by thyroiditis, while 38.9% of the non-IONM group was accompanied by thyroiditis (*p* = 0.676). Thus, two groups were not significantly different in age, cancer proportion, and accompanying thyroiditis, while gender and nodule size significantly differed between groups. No vocal cord palsy, either transient or permanent, was detected in any reviewed cases.

**Figure 3:**
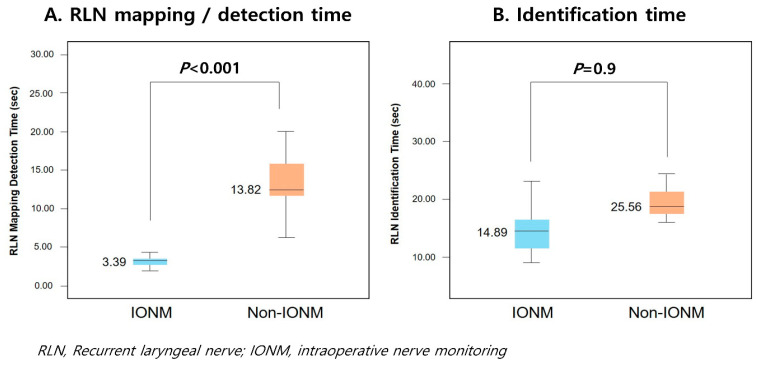
(**A**) Comparison of RLN mapping/detection time and (**B**) identification time between IONM and non-IONM groups.

In comparison of RLN detection and confirmation time between the IONM and non-IONM groups, RLN detection time was 10.43 s shorter (*p* < 0.001), and confirmation time was 10.67 s shorter (*p* = 0.09) in the IONM group than in the non-IONM group. Detection time and confirmation time were measured using the same LCT approach for all cases in both groups (Figure 3, Table 2).

To localize the RLN, we divided the triangle into four sections, allocated the numbers for each section (Figure 2a), and quantified which section had the most cases. As categorized by anatomical location of the nerve in LCT, most of the right lobe RLNs (*n* = 15) were on Section 1 (*n* = 8, 53%) followed by Section 2 (*n* = 5, 30%), while left lobe RLNs (*n* = 16) were primarily located in Section 1 (*n* = 13, 81%) (Table 3).

## 4. Discussions

Preserving the RLN is the most critical aspect of thyroidectomy. Failure to preserve the RLN can result in life-changing adverse events that can be permanent or transient, including voice changes, temporary airway problems, and permanent tracheostomy in worst case scenarios [2]. Various anatomical approaches and electronic devices are used to locate the RLN and prevent injury [1]. Anatomical definitions for location of the RLN have been proposed in many prior studies, but these definitions are not straightforward and significantly vary between individuals, so their use cannot be standardized [18,19,20,21]. In the present study, we utilized a new anatomical definition, the LCT, which is straightforward and broadly applicable, so can be put into standardized use.

Nodule size was significantly higher in the IONM group than in the non-IONM group, as this was not a randomized prospective study. Nodule size is positively correlated with the risk of RLN injury, so larger nodule size likely influenced the decision for IONM usage to avoid RLN injury. Therefore, this bias likely contributed to the larger nodule size in the IONM group relative to non-IONM.

Despite the fact that nodule size was bigger in the IONM group, detection and identification times were approximately 10 s faster in the IONM group than in the non-IONM group. Although 10 s may not seem a huge measure, this short but significant difference between two groups is still meaningful as all the cases were performed by a single surgeon who has performed high volume of surgeries with skilled techniques, implying the possibility of longer time difference to other surgeons, especially the novices, helping them to perform confident and apt thyroid surgeries in the field. Thus, as suggested by prior studies, IONM allowed faster detection of the RLN during thyroid surgery [9]. All surgeries for both the IONM and non-IONM groups were performed by a single surgeon, and the LCT approach was used in all surgeries.

The LCT is defined as a triangle comprised of lower central neck compartments, including the common carotid artery and lower pole of thyroid gland. We hypothesized that the RLN most commonly ran through the middle of the LCT, which was evaluated in the present study. The result supported the hypothesis that in most cases, the RLN ran in the middle of the LCT for both the right and left lobes of the thyroid. To utilize the LCT in thyroidectomy, accurate anatomical exposure of landmarks, especially appropriate exposure of the common carotid artery, is important. LCT is formed when the thyroid lobe is properly retracted, so the operators may need some practice to retract the thyroid lobe to form LCT as shown in Figure 1. Some endocrine surgeons follow the contour of the thyroid capsule, referred to as ‘capsular dissection’, which frequently does not allow proper exposure of the LCT landmarks [23]. Moreover, the difference of LCT from previously renowned triangles such as Beahr’s triangle is that the identification of the triangle happens prior to the identification of RLNs. In Beahr’s triangle, the identification of RLN should be precedent so that the triangle would be exposed for confirmation. Unlike Beahr’ triangle, the LCT is a triangle consisting of well-defined already-constructed structures by nature that practically guide the practitioners to the actual field of identification of RLNs leading to a safe and confident surgery without nerve injuries.

In the present study, the RLN did not run through the uppermost part of the thyroid lobe in any cases, which is section ‘4’ in Figure 2b. It would be hasty to conclude that the RLN never runs in the uppermost part of the LCT due to the relatively small sample size in the present study. There still is a possibility of RLN found in the uppermost part of LCT as RLNs can be found in various position especially when the tumor is huge or when there are many enlarged lymph nodes according to the experiences. However, the study established the trend that the RLN primarily ran in the middle of the LCT. The same trend was seen in both the IONM and non-IONM groups, implying that the LCT approach was helpful in identifying the RLN regardless of IONM use. However, further studies with larger sample sizes are needed to confirm the anatomical findings of the study.

Although only open thyroidectomy cases were considered in the present study, further studies in other surgical modalities will determine whether the LCT is efficient for RLN detection in these contexts. Because endoscopic thyroidectomy using robotics is an increasingly popular alternative to open thyroidectomy, a subsequent study evaluating the use of LCT and IONM with this modality would be helpful in further standardizing surgical protocols for RLN preservation during thyroidectomy [24,25,26].

IONM is an important device for assistance in thyroidectomy, but incorporation of IONM in thyroid surgery widely varies. Some endocrine surgeons use IONM to guide RLN detection after crude dissection, while other surgeons use IONM to confirm the RLN after fine anatomical dissection [5,7,10,22]. However, regardless of how IONM is used, LCT provides a promising anatomical exposure and a good view of the RLN to make IONM use meaningful.

The present study has some limitations. The study is inherently limited by its retrospective design and small sample size done by only one surgeon. To further confirm the findings presented here, a large randomized prospective study is under preparation.

## 5. Conclusions

In conclusion, the present study clearly defined the LCT for the first time, which can be used to detect the RLN in thyroidectomy, particularly with use of IONM as a surgical aid. The LCT can be used as a standardized approach to locate the RLN, which cannot be consistently located between cases using other anatomical approaches.

## Figures and Tables

**Table 1:** Demographic characteristics of IONM ^*^ and non-IONM ^*^ groups.

Characteristics	Non-IONM * (*n* = 18, 45%)	IONM * (*n* = 22, 55%)	*p* Value
Age (year)	51.78 ± 13.42	51.73 ± 12.37	0.990
Gender, Male	4 (22.2%)	5 (22.7%)	<0.001
Cancer	17 (94.4%)	19 (86.4%)	0.421
Nodule size (cm)	1.21 ± 0.98	2.22 ± 1.56	0.037
Thyroiditis on pathology	7 (38.9%)	10 (45.5%)	0.676

* IONM, Intraoperative nerve monitoring.

**Table 2:** Comparison of mapping/detection and identification times of the RLN ^†^ between IONM * and non-IONM * groups.

	Mapping/Detection (s)	Identification (s)	*p*
IONM	3.3936 ± 1.55	14.8932 ± 7.33	<0.001
non-IONM	13.8189 ± 8.60	25.5589 ± 14.12	0.009

† RLN, Recurrent laryngeal nerve; * IONM, intraoperative nerve monitoring.

**Table 3:** Proportion of RLN ^†^ for LCT ^‡^ sections (allocated in Figure 2b) on right and left lobes.

**Right Lobes (*n* = 20)**	non-IONM *	IONM *	**Total**
1	3 (37.5%)	8 (72.7%)	11 (55.0%)
2	3 (37.5%)	3 (27.3%)	6 (30.0%)
3	2 (25.0%)	0 (0.0%)	2 (10.0%)
4	0 (0.0%)	0 (0.0%)	0 (0.0%)
**Left Lobes (*n* = 20)**	non-IONM *	IONM *	**Total**
1	9 (90.0%)	9 (81.8%)	18 (85.7%)
2	0 (0.0%)	2 (18.2%)	2 (9.5%)
3	1 (10.0%)	0 (0.0%)	1 (4.8%)
4	0 (0.0%)	0 (0.0%)	0 (0.0%)

† RLN, Recurrent laryngeal nerve; ‡ LCT, lower central triangle; * IONM, intraoperative nerve monitoring.
